# Congenital Sensorineural Deafness in Australian Stumpy-Tail Cattle Dogs Is an Autosomal Recessive Trait That Maps to CFA10

**DOI:** 10.1371/journal.pone.0013364

**Published:** 2010-10-12

**Authors:** Susan Sommerlad, Allan F. McRae, Brenda McDonald, Isobel Johnstone, Leigh Cuttell, Jennifer M. Seddon, Caroline A. O'Leary

**Affiliations:** 1 School of Veterinary Science, The University of Queensland, Gatton, Queensland, Australia; 2 Centre for Companion Animal Health, The University of Queensland, Brisbane, Queensland, Australia; 3 Queensland Statistical Genetics, Queensland Institute of Medical Research, Brisbane, Queensland, Australia; New Mexico State University, United States of America

## Abstract

**Background:**

Congenital sensorineural deafness is an inherited condition found in many dog breeds, including Australian Stumpy-tail Cattle Dogs (ASCD). This deafness is evident in young pups and may affect one ear (unilateral) or both ears (bilateral). The genetic locus/loci involved is unknown for all dog breeds. The aims of this study were to determine incidence, inheritance mechanism, and possible association of congenital sensorineural deafness with coat colour in ASCD and to identify the genetic locus underpinning this disease.

**Methodology/Principal Findings:**

A total of 315 ASCD were tested for sensorineural deafness using the brain stem auditory evoked response (BAER) test. Disease penetrance was estimated directly, using the ratio of unilaterally to bilaterally deaf dogs, and segregation analysis was performed using Mendel. A complete genome screen was undertaken using 325 microsatellites spread throughout the genome, on a pedigree of 50 BAER tested ASCD in which deafness was segregating. Fifty-six dogs (17.8%) were deaf, with 17 bilaterally and 39 unilaterally deaf. Unilaterally deaf dogs showed no significant left/right bias (p = 0.19) and no significant difference was observed in frequencies between the sexes (p = 0.18). Penetrance of deafness was estimated as 0.72. Testing the association of red/blue coat colour and deafness without accounting for pedigree structure showed that red dogs were 1.8 times more likely to be deaf (p = 0.045). The within family association between red/blue coat colour and deafness was strongly significant (p = 0.00036), with red coat colour segregating more frequently with deafness (COR = 0.48). The relationship between deafness and coat speckling approached significance (p = 0.07), with the lack of statistical significance possibly due to only four families co-segregating for both deafness and speckling. The deafness phenotype was mapped to CFA10 (maximum linkage peak on CFA10 −log10 p-value = 3.64), as was both coat colour and speckling. Fine mapping was then performed on 45 of these 50 dogs and a further 48 dogs (n = 93). Sequencing candidate gene *Sox10* in 6 hearing ASCD, 2 unilaterally deaf ASCD and 2 bilaterally deaf ASCD did not reveal any disease-associated mutations.

**Conclusions:**

Deafness in ASCD is an incompletely penetrant autosomal recessive inherited disease that maps to CFA10.

## Introduction

Congenital sensorineural deafness is usually an inherited form of deafness, and has been reported in over 80 breeds of dogs. The incidence of congenital deafness in ASCD is unknown. However, in the United States the incidence is reported as 14.5% in the separate, but likely related breed, the Australian Cattle Dog [1].

Congenital sensorineural deafness in most dog breeds is due to cochleosaccular degeneration commencing in the first 4 weeks of life [1], [2], [3], [4]. Histologic studies show the inner ear develops normally in most deaf puppies for 1–4 weeks postnatally, but then either cochleosaccular or neuroepithelial degeneration develops [1], [2], [3], [4], [5]. A review of studies of histological cochlear changes seen in deaf Dalmatians show common findings to be atrophy of the stria vascularis, collapse of the cochlear duct and degeneration of the organ of Corti and tectorial membrane [6].

In congenital cochleosaccular deafness, strial degeneration is associated with the absence of pigment producing cells or melanocytes [7]. These melanocytes are vital for strial survival [8]–[11], which in turn is required to maintain a suitable environment for cochlear hair cells [12]. This connection between melanocytes and cochlear function may explain the association between lack of pigmentation and deafness reported in many breeds [1], [13]–[15]. Specifically, this includes a lack or dilution of coat and eye pigmentation (as in the merle Border Collie), and the piebald or white colouration (as in the Bull Terrier and Dalmatian) [1], [13]–[15]. A study in Australian Cattle Dogs in 2004 found no association between pigmentation and deafness in 293 animals [1], but no work has been published on the ASCD.

While the inheritance mechanism for congenital deafness in dogs has not been definitively confirmed in any large studies, there have been several reports on the possible inheritance mechanism of this disorder in various breeds [16]–[19]. The large variation within and between breeds in the reported mode of inheritance may be due to heterogeneity in the genetic mechanism among breeds or to the inconsistent use of the gold standard diagnostic method, the brain stem auditory evoked response (BAER) test, to determine phenotype. Many inheritance studies have been performed in Dalmatians, with results reported as involving two recessive genes [20], an autosomal pleiotropic recessive gene with incomplete penetrance [15] (although this study did not use BAER testing), a major recessive gene with a polygenic component [14] and polygenic inheritance [17]. More recently, Muhle *et al.* (2002) suggested a monogenic inheritance model with incomplete penetrance fitted their data better than the polygenic model [18]. A German study in Dalmatians reported a major recessive gene causing deafness with eye colour as a covariate best explained their data [19]. This latter study also found that while deafness was associated with pigmentation, in many cases deafness could be attributed to a major recessive gene not linked with coat colour or pigmentation.

Genetic studies screening the whole genome are increasingly being used to investigate inherited diseases of dogs. Even using a relatively small numbers of dogs, candidate loci have been identified in diseases involving either a single locus or several loci [21]. However, an analysis restricted to a single breed and a well diagnosed phenotype are prerequisites in investigating a disorder such as sensorineural deafness with apparent heterogeneity among, and perhaps within, breeds. Thus, the aim of this study was to determine the incidence of congenital sensorineural deafness in ASCD in Australia, assess its inheritance mechanism, and investigate its possible association with coat colour in 315 BAER tested animals. Furthermore, using a pedigree of 50 ASCD in which deafness was segregating, we aimed to identify regions of the genome associated with congenital deafness in the ASCD by performing a complete genome screen.

## Results

### Penetrance and inheritance of deafness

The incidence of congenital sensorineural deafness in the ASCD was 17.8% (56 deaf from 315 tested). Of these, 17 were bilaterally deaf and 39 unilaterally deaf. Of the unilaterally deaf animals, 15 were deaf in the left ear, 23 in the right ear and one had the ear unrecorded, providing no significant evidence for a left/right asymmetry (p = 0.19). The ratio of males to females (23 to 33) was not significantly different to that expected by chance (Fisher's exact test, p = 0.18). Within each sex, the ratio of bilaterally to unilaterally deaf dogs did not differ significantly (7 bilateral to 16 unilateral for males, 10 bilateral to 23 unilateral for females; Fisher's exact test, p = 1). This indicates that the penetrance of deafness does not act in a sex specific manner.

Directly estimating the penetrance of the deafness mutation gave a probability that an individual ear was deaf in a dog with the homozygous deaf genotype of 0.47. Thus, the probability that a dog with the deafness genotype would be deaf in both ears is estimated as 0.22, deaf in one ear as 0.50 and not deaf in either ear as 0.28. This is consistent with the two matings between unilaterally deaf dogs in this pedigree which produced 3 out of 8 and 2 out of 4 puppies with normal hearing.

Examination of the pedigree (Figure S1) suggested an autosomal recessive inheritance mechanism, and this was consistent with segregation analysis. With the directly estimated 72% penetrance, an observed 56 deaf dogs out of 315, and assuming a recessive mode of inheritance, the proportion of dogs that are homozygous for the deaf allele was estimated to be 0.25 ( = 56/0.72/315). Assuming Hardy-Weinberg equilibrium at this locus, the frequency of the deaf allele (the square root of that proportion) would be estimated to be 0.50. However, it is likely that this locus is being actively selected against in the population, which would have the effect of increasing the ratio of heterozygous to homozygous carriers of the locus and resulting in 0.50 being an underestimate of the actual allele frequency.

### Association of deafness with coat colour and some patterns

The relationship between deafness and aspects of the dogs' coat colours were tested in two ways. Firstly, taking a direct approach that ignored pedigree structure, there was a modestly significant relationship between red/blue coat colour and deafness (p = 0.045), with red dogs being 1.84 (OR 95% C.I. 0.98 to 3.48) times more likely to be deaf. For both speckling and facial masks, p-values approached significance (0.074 and 0.065 respectively) with speckled dogs being more likely to be deaf (OR = 1.77) and masked animals less likely to be deaf (OR = 0.42).

A second analysis included within-family analysis of the relationship between traits. Of the families who were segregating for deafness, eight also segregated for red/blue coat colour, four for speckling and ten for facial masks. There was a highly significant within-family association between red/blue coat colour and deafness (p = 0.00036), with red coat colour segregating more frequently with deafness (COR = 0.48). Speckling approached significance in its relationship with deafness (p = 0.073), with a speckled coat pattern being inherited more often with deafness (COR = 0.31). Facial masking showed a weakly significant segregation with deafness (COR = −0.28, p = 0.036). However, after correction for the multiple testing on the three traits, only the association between red/blue coat colour and deafness remained significant.

There was a strong relationship between red coat colour and the presence of speckling, with red dogs being approximately thirty times more likely to have speckling than blue dogs (ignoring pedigree structure). There was also strong within-family correlation between coat colour and speckling (COR = 0.8, p<10^−16^). These associations, and the presence of only four families co-segregating for both deafness and speckled pattern, may explain the strongly significant within-family test for the relationship between red/blue coat colour and deafness but not for speckling and deafness.

### Genome wide linkage scan links deafness and coat characteristics to CFA10

**Figure 1:**
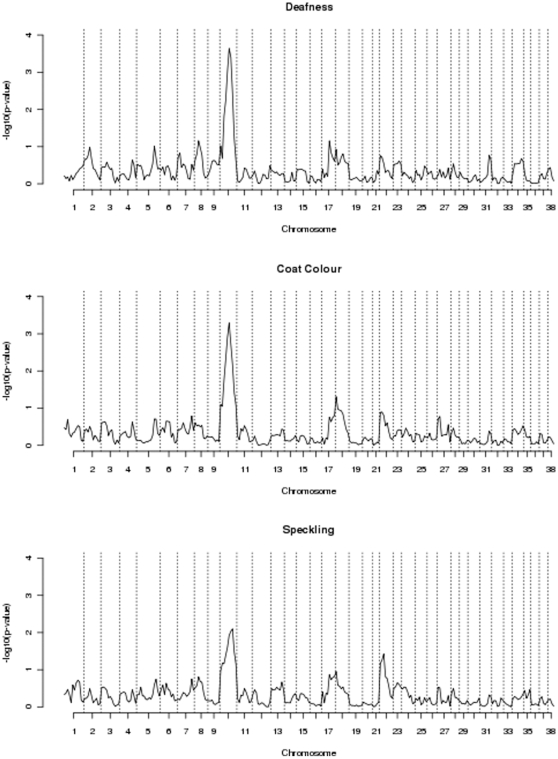
Non-parametric linkage for congenital sensorineural deafness in Australian Stumpy-tail Cattle Dogs to CFA10. Results of the non-parametric linkage scan for congenital sensorineural deafness in Australian Stumpy-tail Cattle Dogs. The maximum linkage peak was found on CFA10 (−log10 p-value = 3.64) at marker ZUBECA1.

**Figure 2:**
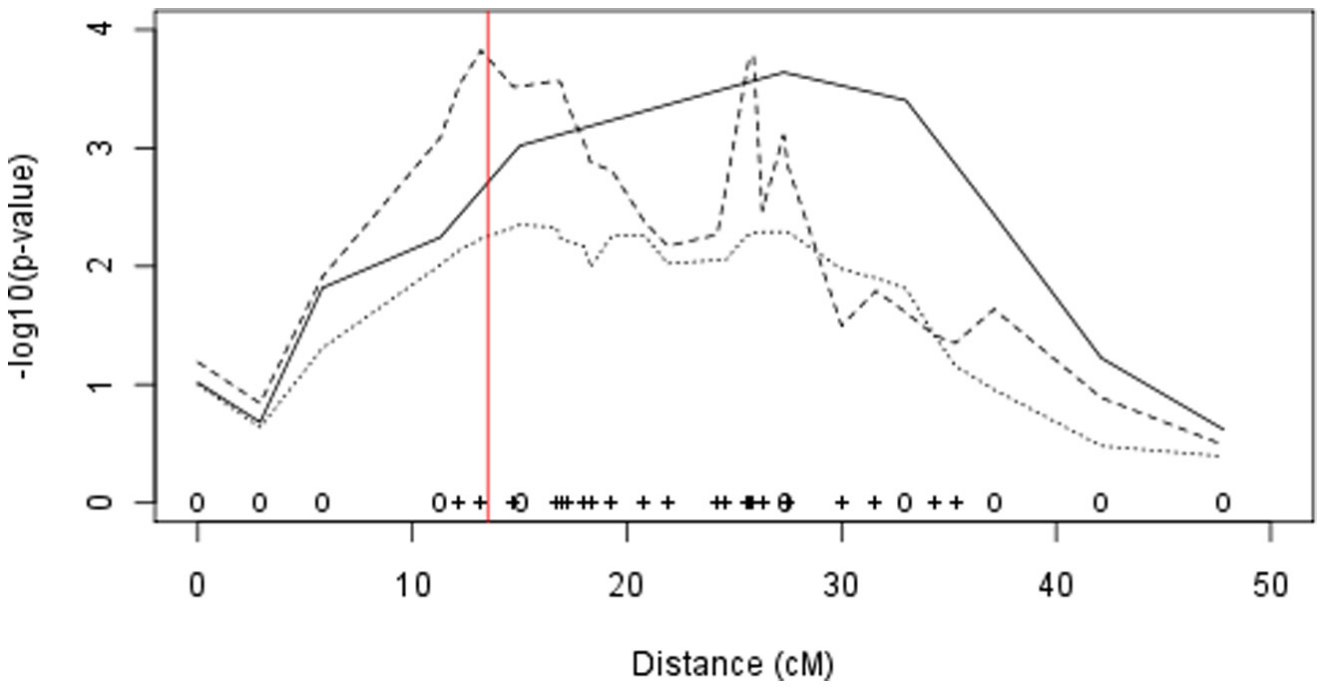
Non-parametric linkage for congenital sensorineural deafness in Australian Stumpy-tail Cattle Dogs to CFA10 between marker FH4081 and 10.05cM. Results of the non-parametric linkage scan for congenital sensorineural deafness in Australian Stumpy-tail Cattle Dogs for chromosome 10. From the genome-scan (solid line), the approximate confidence interval for the location of the underlying genetic variant covers much of the chromosome. The original linkage result is given by the solid line, with the dashed and dotted lines representing the results after the addition of extra markers then additional dogs respectively. Marker positions are indicated at the bottom of the graph (o = marker used in original scan, + = additional marker). The position of *Sox10* is indicated by the vertical line.

The results of the non-parametric linkage scan for deafness shown in Figure 1 have a maximum linkage peak on CFA10 (−log_10_ *p*-value = 3.64) at marker ZUBECA1. However, the approximate confidence interval for the location of the underlying genetic variant covered much of the chromosome. No other chromosome had a test-statistic above 2.0. The addition of extra markers on CFA10 reduced the location confidence interval to between marker FH34081 and 10.05 cM (Figure 2). The map used was (www.vgl.ucdavis.edu/dogset/DOGSET_Markers_Interpolated.xls). Similarly, the highest linkage peaks for both coat colour and speckling were mapped to this chromosome.

The genotyping of an extra 50 BAER tested individuals in the pedigree resulted in a drop in the test statistics across the chromosome, with the most significant marker providing a −log_10_ *p*-value of 2.35. Despite the reduction in the test-statistic, CFA10 remained the only chromosome in the genome containing a test-statistic greater than two. While the reason for this drop remains unclear, potential explanations are unidentified pedigree errors introduced with the additional animals, phenotype mis-specification or due to the limitations of the analysis software with very tightly linked markers. Such pedigree errors were difficult to detect with a single chromosome of genotypes due to the combination of low marker diversity and high relatedness between animals. The marker data supported this theory as they showed slightly higher genotype inconsistencies in the additional animals (data not shown).

### Sequencing of the candidate gene *Sox10*

The gene *Sox10*, a candidate gene involved in pigmentation and in deafness in other species and present on CFA10, was sequenced in unilateral, bilateral deaf and hearing dogs. Eighteen SNPs were identified in gDNA in exon and flanking sequences of *Sox10* in Australian Stumpy-tail Cattle dogs, however none were clearly associated with the deafness phenotype (Table 1).

**Table 1:** No association was evident between *Sox10* polymorphisms in gDNA from hearing, unilateral and bilaterally deaf Australian Stumpy-tail Cattle Dogs.

Location	Hearing red	Hearing blue	Hearing blue speckled	Hearing blue	Hearing red speckled	Hearing red speckled	Deaf left red speckle	Deaf right red	Bilateral deaf blue	Bilateral deaf red
66 bp before exon 1	T	T	T	T	T	T	T	T	W	T
44 bp after exon 1	G	R	G	R	No seq	A	G	R	No seq	G
158 inside exon 2	G	No seq	A	G	No seq	G	G	G	G	G
179 bp inside exon 2	M	No seq	C	C	No seq	C	C	C	C	C
65 bp after exon 2	R	G	G	R	R	G	R	R	G	R
79 bp after exon 3	del	No seq	del	No seq	del	A	del	No seq	del	del
122 bp after exon 3	T	No seq	K	No seq	T	T	No seq	No seq	T	T
304 bp prior to exon 4	7A	No seq	7A	No seq	7A	6A	7A	No seq	7A	7A
122 bp prior to exon 4	G	No seq	G	No seq	G	G	G	No seq	G	R
11bp within exon 4	G	No seq	G	No seq	G	G	G	No seq	R	R
98 bp after exon 4	C	No seq	C	No seq	Y	C	No seq	No seq	Y	Y
164/5 bp after exon 4	del	No seq	G	No seq	del	A	G	No seq	A	G
165 bp after exon 4	T	No seq	G	No seq	T	G	T	No seq	G	G
137 bp before exon 5	Del	del	del	del	del	del	del	del	del	del
134 bp before exon 5	Del	del	del	del	del	del	del	del	del	del
125 bp before exon 5	A	del	del	del	del	del	A	del	A	A
20 bp before exon 5	Y	T	T	Y	Y	T	Y	Y	T	Y
91 bp after exon 5	Del	Del	del	Del	Del	No seq	Del	Del	Del	Del

Bp base pair; T = Thymine; W = A or T; G = Guanine; R = A or G; A = Adenine; C = Cytosine; M = A or C; Del = Deletion; No seq = No sequence available; K = G or T; Y C or T.

## Discussion

### Penetrance and inheritance mechanism for deafness

In this study, the inheritance of deafness in the Australian Stumpy-tail Cattle Dog was consistent with an autosomal recessive pattern. Recent reports on inherited deafness in Dalmatians also implicate a monogenic [18] or a major recessive gene along with other loci [19]. In a Californian study in Dalmatians, there was evidence for an association between a single major locus with a role in auditory development and deafness. However, heritability was estimated at 0.21, making this locus not the only one responsible for deafness [14]. A subsequent study in 2004 [7], found that the evidence for a single major gene affecting the condition was not conclusive despite a heritability estimate for the phenotype of 0.73, and that this model did not completely explain the inheritance of inherited deafness in this breed. Interestingly, 80% of nonsyndromic prelingual deafness in humans is estimated to be autosomal recessive, with 20% autosomal dominant, 1% X-linked and <1% mitochondrial [22]. Purebred dogs often originate from small numbers of individuals and animals are often bred to close relatives. While this pattern of breeding does not create disease alleles, it does result in high levels of homozygosity, and hence autosomal recessive genetic diseases are common in purebred dogs.

In this study, the direct estimation of the penetrance of deafness, using the ratio of unilateral to bilaterally deaf dogs, was 72%. Thus, there is an estimated 28% chance that a dog homozygous for the deafness-associated mutation was not deaf in either ear. Consequently, families resulting from unilateral deaf×unilateral deaf matings were expected to contain non-deaf puppies, as not all dogs that are homozygous for a mutation causing deafness would be deaf. This occurred in this study and has been reported previously in other breeds [7].

This direct estimate of the penetrance of deafness was calculated assuming that each ear independently developed deafness, and thus whether a carrier was unilaterally or bilaterally deaf occurred at random. Altered premelanocyte migration, maturation or function is likely to be part of the mechanism for deafness in this breed, given the association between coat colour and markings and deafness. Similarly in Dalmatians, an association may occur between congenital sensorineural deafness and a lack of pigmentation and melanocytes in the skin and inner ear [23]. In congenital sensorineural deafness in Dalmatians, as in mouse mutants and white cats, the lack of melanocytes in the stria vascularis occurs in the embryo, and this lack of strial melanocytes leads to dysfunction of potassium channels, subsequent unrecordable endocochlear potentials and consequent severe deafness in the affected ear [23]. Thus, it is likely that the presence or absence of strial melanocytes in deaf ears in breeds with the common cochleosaccular pigment related deafness is a random event in the embryo, and equally likely to occur in either ear in deaf animals.

### Genome screen deafness identifies locus on CFA10

Results of this linkage study clearly identified a single linkage peak for the congenital deafness phenotype in ASCD on CFA10. No other chromosomes demonstrated significant linkage to deafness. Interestingly, this region of CFA10 contains a good candidate gene, Sry-related Hmg-box gene 10 or *Sox10*. This gene is involved in Waardenburg-Shah syndrome in humans which includes deafness and hypopigmentation in its phenotype (http://www.ncbi.nlm.nih.gov/entrez/dispomim.cgi?id=602229). Further, *Sox10* is expressed in foetal brain in the rodent [24], [25]. *Sox10* interacts with *Mitf* [26], and the latter has been identified as a cause of white spotting [21] and extreme white spotting in some breeds of dogs including the Dalmatian and Bull Terrier [27].

*Sox10* acts as an activator of *Mitf*, which controls development and postnatal survival of melanocytes. A *Sox10* mouse mutant reduces wildtype *Sox10* induction of *Mitf* [26], suggesting hypopigmentation in this model is due to disrupted function of *Mitf*. *Sox10* is also vital for neural crest development. Thus, a *Sox10* mutation in ASCD could change coat colour, possibly cause white speckling and deafness through a lack of melanocyte function in the inner ear and altered neural crest development. However, sequencing 700bp 5′ to the start codon, all predicted exons and at least 100bp of each intron in gDNA did not find any obviously disease-associated variants. Sequencing *Sox10* mRNA and investigating regulatory mutations from stria vascularis could be useful in further investigating the possible role of this gene in this phenotype.

Of course, *Sox10* is by no means the only gene under the region indicated by the linkage peak. Another plausible candidate is *MYH9*, which has variants known to cause deafness in humans [28]. However, it is not known to interact with melanocytes, a key component of the biological mechanism of this disease.

### Relationship to coat colour and some coat patterns

In this study, only weak evidence for the involvement of speckling, that did not survive the correction for multiple testing, was found. The lack of a strongly significant relationship between deafness and the forms of white spotting, or lack of coat pigmentation, evaluated in this study was unexpected given the likely biological mechanism for the disease. However, the weak evidence for the relationship between speckling and deafness in this study is likely to be due to the lack of power afforded by only four families co-segregating for both deafness and speckling in the sample tested.

The ASCD is born with a white coat that darkens at about 3 weeks of age and may start to show a diffuse speckling pattern. It has been suggested that gene/s producing a roaning effect, may be involved in the speckling pattern seen in the Australian Cattle Dog coat [29]. However, the S or white spotting locus has recently been identified as *Mitf* on CFA20 [21]. While the *Mitf* locus has been associated with deafness in breeds with white coat spotting in one recent study [30], the genome screen in our study suggests that the locus responsible for speckling in ASCD, if it is associated with deafness in this breed, may be on CFA10.

The origin of deafness in ASCD is unknown, and indeed there is some disagreement about the breed's origins. The ASCD is a uniquely Australian breed reported by dog breeders to have a similar ancestry to the Australian Cattle Dog, which descended from Halls Heelers, a dog produced from crossing the Northumberland Blue Merle Drovers dogs with Dingos [31]. Historically, some dog breeders such as Kaleski, also believed Dalmatian and Australian Kelpies were included in the breeding of the Australian Cattle Dog [32]. Bull Terrier infusions may have also have occurred [33]. Others believe that the ASCD developed from crossing first the Black Bobtail Smithfield and the Dingo, producing the Red Bobtail, and then crossing with the Smooth-haired Blue Merle Collie. However, congenital deafness in the ASCD is not easy to reconcile with an origin from the Smooth-haired Blue Merle Collie as the ASCD does not show the merle colouration or white head patches that are associated with an increased incidence of deafness in other breeds such as the Border Collie [13]. Similarly, the origin of congenital deafness in the ASCD is not easy to reconcile with the white base coat of the Bull Terrier that is associated with congenital deafness in this breed. This white coat colour may be due to alleles of the *Mitf* gene [27] on CFA20. However, in the current study the speckled coat colour in the ASTCD and deafness appears to be on CFA10.

However, the white base coat in the Dalmatian (with late appearance of dark spots) that is associated with deafness [1], may be similar in origin to the white birth coat (and later appearance of speckled and red/blue coat) of the ASCD. Thus, if the origin of deafness in ASCD was from Dalmatians, this could be consistent with association between deafness in ASCD and the ticking or speckled phenotype.

Interestingly in this study, a highly significant relationship between coat colour and deafness was observed, with red dogs being approximately twice as likely to be deaf than blue dogs. This is a new finding, as previously reported associations between deafness and pigmentation have not included red coat colour [1]. The reason for this association is unclear, however combining the evidence for the linkage between red coat colour and deafness with the evidence for linkage between red coat colour and speckling, these three loci may in fact form a tightly linked cluster in the ASCD.

However, if the red coat colour in ASCD is in fact linked to deafness and speckled coat colour in the ASCD, none of the currently reported loci causing red coat colour, such as the *Mc1r* locus [29] on CFA5, are the cause of red coat colouration in the ASCD. Interestingly, masks have been reported to be due to an alternate allele of the *Mc1r* locus, termed Em [29]. The Em allele only needs to be present as a single copy to produce the mask, however the black mask is not visible on blue dogs [29] and the mask in the red ASCD in this study was red not black. It is unknown whether the Em allele is responsible for the facial mask in the ASCD. So while the weak negative relationship found in this study between facial masks and deafness may support this locus as one of possible interest in congenital deafness in the ASCD, no genetic evidence has been published to confirm this. Similarly, neither the second region of the genome associated with red coat colour in dog breeds, the dominantly acting Ay allele at the Agouti locus (ASIP) on CFA24 [29], or the third region containing the K locus on CFA16, which may also cause a red coat indistinguishable from that caused by *Mc1r* recessive red allele [34], are implicated in red coat and deafness in the ASCD.

Another possible explanation for the association between red coat, speckled coat and deafness in ASCD is that the speckled phenotype may be more highly penetrant in red dogs, which would allow the speckled locus to be unlinked to both the coat colour and deafness loci. These hypotheses could be separated by performing tests of association between deafness and speckling within each coat colour, but would require a larger pedigree.

In summary, congenital sensorineural deafness in the ASCD is an autosomal recessive disease trait with incomplete penetrance. The locus for this disease, the red coat and likely the speckled coat pattern, all map to CFA10 in the ASCD.

## Materials and Methods

### Diagnosis of deafness

Three hundred and fifteen ASCD were examined using BAER testing. Animals under 6 months of age were sedated using 0.05 mg/kg acepromazine (Delvet Ltd, Powers Rd, Seven Hills, NSW Australia) and pethidine HCL 0.5 mg/kg subcutaneously (Hameln Pharmaceuticals Langes Feld 13, Hameln, Germany). Healthy young adult animals over 6 months of age were sedated with medetomidine HCl 5 µg/kg intravenously (Orion Pharma Espoo, Finland). Sedation was reversed after testing with atipamezole HCL 25 µg/kg subcutaneously (Orion Pharma, Espoo, Finland). Animals over five years of age underwent a complete blood count and serum biochemistry health profile and then were tested under a general anaesthetic induced with alfaxaone 1–2 mg/kg intravenously (Jurox Ltd 85 Gardiners Rd Rutherford NSW 2320 Australia) and maintained by intubation administration of isoflurane 1–2% (Bomac Animal Health, 8 Appollo Ave, West Pymble, NSW 2073 Australia) and oxygen.

The BAER testing was performed by two audiologically trained veterinarians (authors Sue Sommerlad and Isobel Johnstone) using an agreed protocol and a Medelec Sapphire 2ME testing system. This method used an alternating click signal of between 2 KHz and 4 KHz at decibel (dB) levels ranging between 30 and 90 dB sound pressure level. The electrode array used leads with 12 mm stainless steel subdermal electrodes. These were placed subcutaneously with the reference lead at the cranial vertex, the recording lead just rostral to the base of the tragus and the ground lead midline in the cranial cervical region. The recorded response was a summation of the response to 1024 clicks delivered at 11 clicks per second and consisted of at least five wave forms, representing stages in the auditory pathway between the auditory nerve and the brain stem in normal dogs [35], [36], or no waveforms in deaf ear/s. White noise was delivered to the other ear simultaneously at a level 30 dB lower than the stimulus level. Deafness was unilateral or bilateral and was absolute in the affected ear [16].

### Coat colour

Pedigrees were collected for all dogs. Details of the animals' coat colour and markings, which were on either a red or blue base coat colour (http://www.ankc.org.au/home/breeds_details.asp?bid=203), were recorded. Speckling was used to mean an even distribution of lighter (white) and darker (red or black) hairs all over the body including the undercoat giving a red speckled or blue speckled coat. There could be dark patches on the head, red in the case of the red coat, and black in the blue coat. If these pigmented patches involved one eye or extended over both eyes passing dorso-laterally on the head, they were described as unilateral (one eye) or bilateral masks (both eyes). No dogs were cream, had black body patches, tan marks or blue eyes.

### Estimation of deafness penetrance

A direct estimation of the penetrance of the deafness mutation was calculated using the numbers of unilaterally and bilaterally deaf dogs. Under the assumption that development of deafness is independent in each ear, the probability that an individual ear in a dog with the deafness genotypes is deaf was estimated as
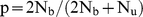
where N_b_ was the number of bilaterally and N_u_ the number of unilaterally deaf dogs. The probability that a dog with the deafness genotype is deaf in both ears was p^2^, deaf in a single ear 2p(1-p) and deaf in neither ear (1-p)^2^.

### Segregation analysis

The pedigree structure of the 315 phenotyped dogs and an additional one generation of 63 unphenotyped and 1 phenotyped ancestors (64 dogs) was used in the segregation analysis and is given in Figure S1. From the phenotyped individuals in this pedigree, there are 223 links between sires and their offspring and 254 links between dams and their offspring. A detailed description of the phenotypic relationships between parents and their offspring is given in Tables 2–4.

**Table 2:** Number of phenotyped sire-offspring pairs/trios in the pedigree used in segregation analyses.

Sire		Offspring Phenotype	
Phenotype	Number	Hearing	Deaf
Hearing	20	167	23
Deaf	3	17	16

**Table 3 pone-0013364-t003:** Number of phenotyped dam-offspring pairs/trios in the pedigree used in segregation analyses.

Dam		Offspring Phenotype	
Phenotype	Number	Hearing	Deaf
Hearing	36	195	38
Deaf	4	14	7

**Table 4:** Number of phenotyped parent-offspring pairs/trios in the pedigree used in segregation analyses.

Phenotype		Number of Matings (involving #sires, #dams)	Offspring Phenotype	
Sire	Dam		Hearing	Deaf
Hearing	Hearing	36 (18, 31)	142	20
Hearing	Deaf	3 (3, 3)	7	1
Deaf	Hearing	5 (3, 5)	9	10
Deaf	Deaf	2 (2, 2)	5	6

For the segregation analysis, deafness was treated as a dichotomous trait, where unilaterally and bilaterally deaf dogs were combined as *affected*. In addition to the 315 dogs tested for deafness, a further one BAER tested dog and 63 non-BAER tested dogs, being the first generation of ancestors, were included in the analysis. Segregation analysis was performed using the “Penetrance Estimation” in Mendel (v8.0.1) [37], using the logit link function. A single major locus was fitted to the pedigree under both genotypic and recessive models with incomplete penetrance. Given the recessive genetic model, an estimate of the deafness allele frequency was obtained directly from the numbers of deaf animals while ignoring pedigree structure.

### Association of coat colour and deafness

Relationship between deafness and the coat attributes of red/blue coat colour, speckling, and facial masks were tested in two ways. Firstly we tested for correlation between deafness and the trait of interest using a Fisher's exact test using all dogs. The advantage of this method is that it does not rely on segregation within families, however it ignores the pedigree structure and thus the estimated effect sizes and p-values may be biased. Hence, a second family-based analysis was performed, which tested for co-segregation of coat traits within families, using an approach similar to extensions of the transmission-disequilibrium-test [38]. (Tables 5–7) Taking a nuclear family that segregates for both deafness and the trait of interest, deafness within each individual was coded as 0 for normal hearing and 1 for deafness. This was then adjusted so that the family mean was zero by subtracting the family mean from the individual's value (example in Table 8). A similar correction was made for the trait of interest. The correlation between the adjusted deafness and the adjusted trait values across all families was then tested for significance.

**Table 5:** Numbers of, and coat colours in, dogs within nuclear family groups (parents and offspring) used to test for within-family tests of genetic correlations between deafness and coat traits.

	Hearing/Red	Hearing/Blue	Deaf/Red	Deaf/Blue
Family 1	4	3	1	0
Family 2	2	2	1	0
Family 3	2	3	1	0
Family 4	1	1	1	0
Family 5	1	16	1	0
Family 6	0	1	1	1
Family 7	0	1	1	0
Family 8	3	2	1	0

**Table 6 pone-0013364-t006:** Numbers of, and coat speckling in, dogs within nuclear family groups (parents and offspring) used to test for within-family tests of genetic correlations between deafness and coat traits.

	Hearing/Plain	Hearing/Speckled	Deaf/Plain	Deaf/Speckled
Family 1	3	1	0	1
Family 2	1	6	0	1
Family 3	16	1	0	1
Family 4	1	0	1	1

**Table 7:** Numbers of, and presence of masking in, dogs within nuclear family groups (parents and offspring) used to test for within-family tests of genetic correlations between deafness and coat traits.

	Hearing/Plain	Hearing/Masked	Deaf/Plain	Deaf/Masked
Family 1	6	1	1	0
Family 2	2	2	1	0
Family 3	2	1	4	0
Family 4	4	0	0	1
Family 5	4	1	1	0
Family 6	0	1	1	0
Family 7	4	1	1	0
Family 8	3	5	1	0
Family 9	1	3	1	0
Family 10	1	1	1	0

**Table 8:** Example coding for the within-family test for association between deafness and speckling.

Individual	Deaf*	Speckled*	Adjusted Deafness	Adjusted Speckling
Dog 1	0	0	−0.5	−0.25
Dog 2	1	0	0.5	−0.25
Dog 3	1	1	0.5	0.75
Dog 4	0	0	−0.5	−0.25
**Mean**	0.5	0.25		

* Coding: 0 = absent, 1 = present.

The adjusted values are calculated by subtracting the family mean from the phenotypic value. The correlation between the adjusted values is a robust test of genetic association between the traits.

### Genome screen

Genomic DNA from all 50 ASCD (7 bilaterally deaf, 6 unilaterally deaf in the left ear, 7 unilaterally in the right ear, 1 unilaterally deaf in unrecorded ear, and 29 normally hearing dogs) was extracted from peripheral blood collected in EDTA using a salting-out extraction method [39].

A panel of 325 microsatellite markers [40] (Table S1) were amplified in multiplex PCR using a M13 fluorescently-labelled protocol [41]. Multiplex optimisation was verified by consistency with singleplex reactions. Each multiplex reaction contained 10 ng of genomic DNA, 5 µl of HotStarTaq Multiplex Mastermix (QIAGEN, Germany), and 0.05–0.3 µM of each forward, reverse primer and M13 primers labelled with 6-fam (Geneworks, Australia), Ned Vic or Pet (ABI, USA), and Q solution (0–2×) (QIAGEN, Germany) in a 10 µl reaction volume. Thermocycling conditions were 95°C for 15 min followed by 35 cycles of 94°C 30 s, 55–65°C 90 s, 72°C 60 s then one cycle of 60°C for 30 min. Fragment separation was carried out on an 3130×l Genetic Analyzer (ABI Biosystems, USA) according to the manufacturer's recommendations. Data analysis was carried out using Genemapper software version 3.7 (ABI Biosystems, USA).

### Analysis of genome screen

Marker inheritances that were incompatible with the pedigree data were removed using PedCheck v1.00 [42]. Four dogs showed consistently large numbers of marker inheritance issues across the genome, indicating either a pedigree error or sample mix-up, and were removed from the analysis. The expected marker order was confirmed using the “flips” option of Cri-Map v2. (http://linkage.rockefeller.edu/soft/cripmap/4). Non-parametric linkage mapping was performed using SimWalk2 v2.91 (http://www.genetics.ucla.edu/software/) using the ENTROPY statistic. For computational tractability, only the genotyped individuals and their first-degree ancestors, giving a total of 78 individuals (46 genotyped) across two families, were included in the analysis pedigree.

### Finescale mapping on CFA10

Following initial analysis that indicated linkage around the ZUBECA1 marker on CFA10, thirty microsatellites were selected from publicly available databases (http://www.vgl.ucdavis.edu/dogset/) in the 25–55Mb region, selecting every 15^th^ microsatellite. A list of primers used is available in Table S2. Microsatellites were amplified in seven optimised multiplex PCR as described above. An additional BAER tested 50 dogs were genotyped at these markers. These primarily represented first-degree relatives to the dogs genotyped for the genome-wide scan. In this analysis a further one dog was removed from the original genotyped 50 dogs, and two were removed from the second 50 genotyped dogs, as they showed consistently large numbers of marker inheritance issues across the genome. The pedigree used for the fine mapping consisted of the 93 genotyped animals and 36 first-degree ancestors across two independent families.

Due to the tight linkage between the additional markers, SimWalk2 had difficulties accurately sampling from the entire sample space. To help rectify this, the analysis was repeated 100 times and the average p-value over all runs was calculated. Repeating this procedure provided QTL profiles that were very similar (data not shown), indicating that the 100 repeats were adequate to achieve good coverage of the sample space. One marker was excluded from the analysis due to a lack of linkage to its expected surrounding markers.

### Sequencing of candidate gene *Sox10*

A candidate gene approach was undertaken once initial linkage from the genome screen was identified to CFA10, and *Sox10* was selected due to its involvement in deafness in humans. *Sox10* exons were predicted by aligning XM_538379.2| PREDICTED: *Canis familiaris* gene similar to SRY (sex determining region Y)-box 10 (LOC481258), with mRNA sequence from Cfa10_WGA3 *Canis familiaris* chromosome 10 genomic contig (genome contig released in May 2005; CanFam2.0). The alignment was performed in Spidey (www.ncbi.nlm.nih.gov/spidey/). Sequencing of the five exons predicted in this gene and over 700bp of the putative promoter sequence 5′ to the start codon, was carried out in 10 of the 50 BAER typed individuals. These 10 included four deaf dogs (2 bilaterally deaf, 2 unilaterally deaf) and 6 dogs with normal hearing. Of the bilaterally deaf dogs, both were blue with no speckling and of the unilaterally deaf dogs, both were red with speckling. The 6 hearing dogs included blue dogs with both speckling and non-speckling and red dogs with speckling (Table 1).

Sequencing of this gene was carried out in eight fragments, and using gDNA. Due to the difficult nature of the *Sox10* sequence (GC content 55–74% for most exons), PCR amplification was undertaken using several different taq polymerases and cycling annealing temperatures (Table S3) and primers from the DOGSET of UC Davis (http://www.vgl.ucdavis.edu/dogset/) with one additional primer designed using Primer3.0 http://frodo.wi.mit.edu/primer3/. DNA sequencing was performed using an ABI 3130×l Genetic Analyzer (Applied Biosystems) with Big Dye 3.0 chemistry, after which sequences were edited and assembled using ChromasPro (Technelysium, Australia). Sequence analysis was performed using MEGA version 4.0 [43].

## Supporting Information

Figure S1 Multigenerational Australian Stumpy-tail Cattle Dog pedigree in which deafness is segregating. Graphical depiction of the multigenerational pedigree used in the segregation analysis. Phenotyped dogs are shaded either white (hearing) or black (deaf), while unphenotyped animals are shaded orange.
(3.39 MB TIF)

Table S1 Summary of marker data using in linkage mapping. For each marker, the number of dogs genotyped, number of observed alleles, observed heterozygosity and estimated polymorphism information content (PIC) is provided. Not that the observed heterozygosity is often lower than the PIC due to inbreeding within the pedigree.
(0.48 MB DOC)

Table S2 List of primers used in fine mapping of the deafness locus on CFA10. Further details of these primers are available at (http://www.vgl.ucdavis.edu/dogset/).
(0.05 MB DOC)

Table S3 PCR primers used for amplification of the Sox10 gene. Primers were from the DOGSET of UC Davis (http://www.vgl.ucdavis.edu/dogset/) with the exception of SOX10_EXO4bmF which was designed using Primer3.0 (http://frodo.wi.mit.edu/primer3/).
(0.06 MB DOC)
