# Supplementary figures and images for: Congenital Sensorineural Deafness in Australian Stumpy-Tail Cattle Dogs Is an Autosomal Recessive Trait That Maps to CFA10
Source: PLoS One. 2010 Oct 12;5(10):e13364. doi: 10.1371/journal.pone.0013364 (PMC2953516; doi:10.1371/journal.pone.0013364)

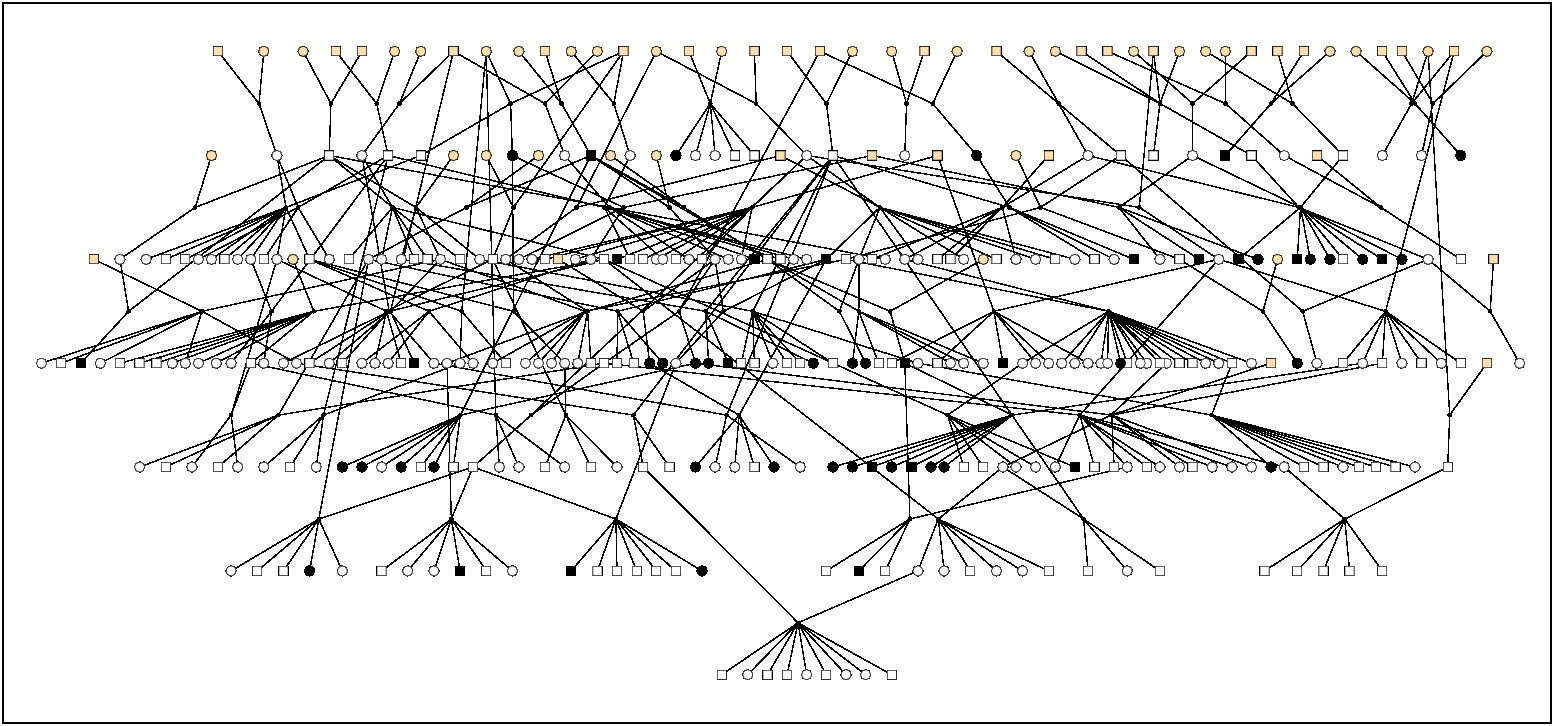

Supplement: Figure S1 — Multigenerational Australian Stumpy-tail Cattle Dog pedigree in which deafness is segregating. Graphical depiction of the multigenerational pedigree used in the segregation analysis. Phenotyped dogs are shaded either white (hearing) or black (deaf), while unphenotyped animals are shaded orange. (3.39 MB TIF) [file pone.0013364.s001.tif]
