# Supplementary material for: Congenital Sensorineural Deafness in Australian Stumpy-Tail Cattle Dogs Is an Autosomal Recessive Trait That Maps to CFA10
Source: PLoS One. 2010 Oct 12;5(10):e13364. doi: 10.1371/journal.pone.0013364 (PMC2953516; doi:10.1371/journal.pone.0013364)
Supplement: Table S1 — Summary of marker data using in linkage mapping. For each marker, the number of dogs genotyped, number of observed alleles, observed heterozygosity and estimated polymorphism information content (PIC) is provided. Not that the observed heterozygosity is often lower than the PIC due to inbreeding within the pedigree. (0.48 MB DOC) [file pone.0013364.s002.doc]

**Supplementary Table 1:** Summary of marker data using in linkage mapping.  For each marker, the number of dogs genotyped, number of observed alleles, observed heterozygosity and estimated polymorphism
information content (PIC) is provided.  Not that the observed heterozygosity is often lower than the PIC due to inbreeding within the pedigree.

| **Marker** | **Chromosome** | **Number of dogs genotyped** | **Number of alleles** | **Microsatellite heterozygosity** | **Microsatellite polymorphism information content** |
| --- | --- | --- | --- | --- | --- |
| C00901 | 1 | 50 | 4 | 0.39 | 0.63 |
| FH2309 | 1 | 48 | 7 | 0.45 | 0.75 |
| FH3325 | 1 | 49 | 6 | 0.41 | 0.78 |
| FH3922 | 1 | 41 | 12 | 0.40 | 0.86 |
| C01.254 | 1 | 47 | 3 | 0.22 | 0.30 |
| FH2294 | 1 | 47 | 7 | 0.38 | 0.71 |
| FH3413 | 1 | 46 | 8 | 0.38 | 0.80 |
| REN112I02 | 1 | 43 | 4 | 0.34 | 0.54 |
| FH2326 | 1 | 45 | 11 | 0.48 | 0.84 |
| FH2663 | 1 | 45 | 9 | 0.31 | 0.60 |
| FH3300 | 1 | 36 | 6 | 0.31 | 0.72 |
| FH3603 | 1 | 44 | 8 | 0.42 | 0.78 |
| C02.609 | 2 | 48 | 2 | 0.22 | 0.35 |
| FH2613 | 2 | 50 | 6 | 0.29 | 0.55 |
| FH3210 | 2 | 50 | 7 | 0.42 | 0.79 |
| REN70M014 | 2 | 50 | 7 | 0.39 | 0.69 |
| C02.342 | 2 | 50 | 4 | 0.38 | 0.56 |
| FH2274 | 2 | 49 | 6 | 0.42 | 0.68 |
| FH2608 | 2 | 43 | 7 | 0.28 | 0.72 |
| FH3965 | 2 | 44 | 4 | 0.30 | 0.60 |
| FH2132 | 2 | 35 | 8 | 0.17 | 0.73 |
| FH2890 | 2 | 50 | 5 | 0.31 | 0.57 |
| FH2302 | 3 | 49 | 9 | 0.43 | 0.81 |
| FH3377 | 3 | 50 | 8 | 0.42 | 0.79 |
| FH3464 | 3 | 50 | 8 | 0.41 | 0.72 |
| C03.629 | 3 | 49 | 5 | 0.39 | 0.74 |
| FH3115 | 3 | 50 | 3 | 0.26 | 0.47 |
| FH3252 | 3 | 46 | 6 | 0.23 | 0.49 |
| FH2145 | 3 | 46 | 8 | 0.41 | 0.80 |
| FH2316 | 3 | 49 | 11 | 0.47 | 0.81 |
| FH3396 | 3 | 42 | 9 | 0.39 | 0.72 |
| REN161A12 | 3 | 50 | 4 | 0.28 | 0.61 |
| REN260I04 | 3 | 48 | 2 | 0.23 | 0.35 |
| AHT103 | 4 | 50 | 4 | 0.23 | 0.46 |
| FH2097 | 4 | 48 | 6 | 0.39 | 0.73 |
| FH2732 | 4 | 50 | 5 | 0.37 | 0.61 |
| FH3310 | 4 | 50 | 7 | 0.27 | 0.59 |
| REN74B12 | 4 | 50 | 3 | 0.34 | 0.48 |
| FH2776 | 4 | 49 | 4 | 0.29 | 0.44 |
| Go7704 | 4 | 45 | 6 | 0.42 | 0.77 |
| REN195B08 | 4 | 50 | 3 | 0.25 | 0.45 |
| REN298N18 | 4 | 49 | 4 | 0.40 | 0.61 |
| REN303C04 | 4 | 49 | 2 | 0.26 | 0.32 |
| CPH14 | 5 | 50 | 5 | 0.43 | 0.75 |
| FH3702 | 5 | 50 | 5 | 0.42 | 0.70 |
| FH3928 | 5 | 49 | 8 | 0.40 | 0.78 |
| FH3978 | 5 | 40 | 10 | 0.44 | 0.84 |
| C05.771 | 5 | 43 | 6 | 0.13 | 0.43 |
| DTR05.8 | 5 | 46 | 14 | 0.47 | 0.89 |
| FH2140 | 5 | 47 | 3 | 0.31 | 0.50 |
| FH3004 | 5 | 47 | 8 | 0.47 | 0.82 |
| FH3089 | 5 | 49 | 5 | 0.32 | 0.66 |
| FH3278 | 5 | 49 | 5 | 0.32 | 0.62 |
| FH3320 | 5 | 49 | 7 | 0.43 | 0.79 |
| REN175P10 | 5 | 49 | 5 | 0.32 | 0.62 |
| REN262G24 | 5 | 39 | 4 | 0.14 | 0.48 |
| REN285I23 | 5 | 29 | 4 | 0.12 | 0.54 |
| FH2164 | 6 | 50 | 8 | 0.41 | 0.66 |
| FH2370 | 6 | 50 | 9 | 0.44 | 0.85 |
| FH3303 | 6 | 49 | 8 | 0.41 | 0.77 |
| FH3933 | 6 | 50 | 6 | 0.36 | 0.65 |
| FH2576 | 6 | 49 | 13 | 0.46 | 0.87 |
| FH2734 | 6 | 49 | 4 | 0.42 | 0.68 |
| REN285H12 | 6 | 49 | 3 | 0.22 | 0.46 |
| FH2119 | 6 | 50 | 6 | 0.44 | 0.74 |
| FH2525 | 6 | 46 | 6 | 0.45 | 0.70 |
| FH2561 | 6 | 47 | 12 | 0.43 | 0.84 |
| FH2860 | 7 | 50 | 3 | 0.35 | 0.49 |
| FH2973 | 7 | 46 | 6 | 0.40 | 0.64 |
| FH3970 | 7 | 37 | 16 | 0.49 | 0.88 |
| FH3972 | 7 | 48 | 9 | 0.43 | 0.74 |
| REN162C04 | 7 | 50 | 5 | 0.30 | 0.53 |
| FH2226 | 7 | 50 | 9 | 0.38 | 0.71 |
| REN143L20 | 7 | 50 | 4 | 0.44 | 0.69 |
| REN286O18 | 7 | 48 | 2 | 0.26 | 0.37 |
| REN97M11 | 7 | 50 | 4 | 0.39 | 0.49 |
| VIA5D10 | 7 | 49 | 7 | 0.40 | 0.67 |
| FH2989 | 8 | 49 | 8 | 0.40 | 0.76 |
| FH3241 | 8 | 49 | 3 | 0.31 | 0.58 |
| FH3316 | 8 | 49 | 5 | 0.35 | 0.66 |
| FH3425 | 8 | 48 | 10 | 0.46 | 0.84 |
| C08.410 | 8 | 50 | 5 | 0.36 | 0.72 |
| C08.618 | 8 | 50 | 5 | 0.41 | 0.63 |
| REN178J05 | 8 | 50 | 5 | 0.39 | 0.64 |
| REN204K13 | 8 | 50 | 2 | 0.25 | 0.32 |
| FH2186 | 9 | 41 | 13 | 0.45 | 0.81 |
| FH2263 | 9 | 43 | 7 | 0.27 | 0.70 |
| FH3835 | 9 | 49 | 5 | 0.29 | 0.67 |
| G06401 | 9 | 50 | 2 | 0.01 | 0.02 |
| FH2885 | 9 | 49 | 5 | 0.36 | 0.63 |
| REN287G01 | 9 | 50 | 4 | 0.21 | 0.47 |
| REN73K24 | 9 | 48 | 3 | 0.27 | 0.41 |
| DTR10.5 | 10 | 47 | 5 | 0.41 | 0.64 |
| FH2293 | 10 | 52 | 8 | 0.40 | 0.76 |
| FH2422 | 10 | 50 | 8 | 0.40 | 0.74 |
| FH2537 | 10 | 52 | 4 | 0.27 | 0.47 |
| FH4081 | 10 | 52 | 6 | 0.17 | 0.36 |
| C10.16 | 10 | 51 | 4 | 0.27 | 0.43 |
| C10.781 | 10 | 51 | 3 | 0.34 | 0.59 |
| FH3381 | 10 | 51 | 8 | 0.34 | 0.63 |
| REN06H21 | 10 | 51 | 2 | 0.19 | 0.29 |
| ZUBECA1 | 10 | 50 | 6 | 0.40 | 0.71 |
| AHT137 | 11 | 50 | 7 | 0.42 | 0.67 |
| C11.868 | 11 | 50 | 4 | 0.30 | 0.47 |
| DGN13 | 11 | 49 | 9 | 0.34 | 0.67 |
| FH2319 | 11 | 45 | 10 | 0.43 | 0.87 |
| FH3203 | 11 | 43 | 5 | 0.16 | 0.39 |
| C11.873 | 11 | 49 | 4 | 0.31 | 0.40 |
| FH2004 | 11 | 49 | 5 | 0.39 | 0.69 |
| FH2019 | 11 | 49 | 4 | 0.17 | 0.33 |
| REN242K04 | 11 | 49 | 3 | 0.31 | 0.39 |
| FH1040 | 12 | 49 | 5 | 0.34 | 0.62 |
| FH3591 | 12 | 49 | 10 | 0.48 | 0.83 |
| FH3711 | 12 | 49 | 7 | 0.48 | 0.77 |
| FH3748 | 12 | 49 | 12 | 0.49 | 0.87 |
| REN94K11 | 12 | 49 | 3 | 0.20 | 0.30 |
| FH2401 | 12 | 45 | 8 | 0.40 | 0.68 |
| G01811 | 12 | 49 | 2 | 0.23 | 0.32 |
| REN258L11 | 12 | 50 | 4 | 0.35 | 0.66 |
| FH2200 | 12 | 39 | 12 | 0.36 | 0.83 |
| REN153O12 | 12 | 50 | 5 | 0.36 | 0.61 |
| REN213F01 | 12 | 50 | 4 | 0.34 | 0.57 |
| FH3619 | 13 | 44 | 7 | 0.43 | 0.74 |
| FH3800 | 13 | 46 | 12 | 0.48 | 0.83 |
| REN120P21 | 13 | 50 | 2 | 0.09 | 0.15 |
| REN227M12 | 13 | 47 | 5 | 0.43 | 0.59 |
| C13.391 | 13 | 50 | 5 | 0.38 | 0.69 |
| DTR13.6 | 13 | 50 | 4 | 0.44 | 0.66 |
| FH2348 | 13 | 49 | 10 | 0.45 | 0.76 |
| FH3494 | 13 | 50 | 7 | 0.36 | 0.58 |
| C14.866 | 14 | 50 | 6 | 0.33 | 0.62 |
| FH2658 | 14 | 50 | 3 | 0.28 | 0.45 |
| FH3285 | 14 | 46 | 13 | 0.43 | 0.85 |
| FH3725 | 14 | 50 | 5 | 0.28 | 0.59 |
| FH2763 | 14 | 50 | 5 | 0.40 | 0.73 |
| FH3951 | 14 | 50 | 10 | 0.45 | 0.84 |
| PEZ10 | 14 | 47 | 6 | 0.35 | 0.70 |
| FH2171 | 15 | 49 | 7 | 0.45 | 0.76 |
| FH2360 | 15 | 43 | 15 | 0.43 | 0.78 |
| FH3802 | 15 | 50 | 2 | 0.30 | 0.35 |
| FH3813 | 15 | 42 | 6 | 0.36 | 0.71 |
| FH3939 | 15 | 48 | 15 | 0.44 | 0.89 |
| FH4012 | 15 | 50 | 6 | 0.38 | 0.59 |
| CPH4 | 15 | 49 | 5 | 0.35 | 0.66 |
| REN06C11 | 15 | 49 | 4 | 0.31 | 0.50 |
| FH2155 | 16 | 45 | 10 | 0.43 | 0.83 |
| FH2175 | 16 | 50 | 8 | 0.41 | 0.76 |
| FH2670 | 16 | 50 | 8 | 0.41 | 0.77 |
| FH3592 | 16 | 42 | 11 | 0.44 | 0.82 |
| REN214L11 | 16 | 47 | 3 | 0.17 | 0.38 |
| REN275L19 | 16 | 49 | 5 | 0.35 | 0.64 |
| REN85N14 | 16 | 49 | 3 | 0.13 | 0.24 |
| FH3369 | 17 | 47 | 8 | 0.43 | 0.76 |
| FH3995 | 17 | 48 | 8 | 0.42 | 0.77 |
| PE28 | 17 | 47 | 6 | 0.28 | 0.58 |
| REN240A05 | 17 | 48 | 3 | 0.08 | 0.15 |
| FH2869 | 17 | 50 | 4 | 0.10 | 0.33 |
| FH3047 | 17 | 50 | 4 | 0.36 | 0.62 |
| FH4023 | 17 | 49 | 6 | 0.40 | 0.65 |
| REN294E18 | 17 | 49 | 5 | 0.37 | 0.69 |
| FH2834 | 18 | 50 | 2 | 0.19 | 0.32 |
| FH3824 | 18 | 49 | 2 | 0.18 | 0.31 |
| FH3944 | 18 | 45 | 15 | 0.43 | 0.86 |
| FH4060 | 18 | 45 | 8 | 0.38 | 0.76 |
| AHT130 | 18 | 50 | 6 | 0.34 | 0.69 |
| FH3815 | 18 | 50 | 10 | 0.39 | 0.78 |
| REN47J11 | 18 | 50 | 5 | 0.19 | 0.42 |
| REN54P11 | 18 | 50 | 6 | 0.26 | 0.53 |
| FH2380 | 19 | 50 | 4 | 0.20 | 0.51 |
| FH3313 | 19 | 49 | 11 | 0.43 | 0.81 |
| FH3491 | 19 | 49 | 11 | 0.47 | 0.85 |
| FH3834 | 19 | 49 | 15 | 0.44 | 0.86 |
| FH2206 | 19 | 45 | 15 | 0.44 | 0.87 |
| *FH3299 | 19 | 50 | 12 | 0.45 | 0.82 |
| FH3969 | 19 | 49 | 9 | 0.38 | 0.73 |
| REN213G21 | 19 | 50 | 2 | 0.06 | 0.11 |
| FH2158 | 20 | 49 | 11 | 0.42 | 0.79 |
| FH2951 | 20 | 45 | 10 | 0.38 | 0.76 |
| REN100J13 | 20 | 50 | 5 | 0.36 | 0.70 |
| REN93E07 | 20 | 50 | 7 | 0.43 | 0.77 |
| AHTK209 | 20 | 47 | 3 | 0.22 | 0.37 |
| REN114M19 | 20 | 50 | 4 | 0.12 | 0.23 |
| FH2233 | 21 | 45 | 13 | 0.47 | 0.88 |
| FH3398 | 21 | 43 | 12 | 0.49 | 0.86 |
| REN118B15 | 21 | 50 | 6 | 0.30 | 0.56 |
| REN37A15 | 21 | 50 | 6 | 0.28 | 0.60 |
| FH3355 | 22 | 47 | 8 | 0.37 | 0.74 |
| REN128E21 | 22 | 50 | 5 | 0.38 | 0.60 |
| REN42F10 | 22 | 50 | 4 | 0.31 | 0.52 |
| REN49F22 | 22 | 50 | 4 | 0.38 | 0.62 |
| C22.279 | 22 | 50 | 5 | 0.40 | 0.68 |
| FH3411 | 22 | 48 | 11 | 0.42 | 0.81 |
| FH3853 | 22 | 50 | 5 | 0.41 | 0.68 |
| REN78I16 | 22 | 50 | 2 | 0.08 | 0.14 |
| FH2508 | 23 | 47 | 6 | 0.34 | 0.63 |
| FH2626 | 23 | 47 | 7 | 0.39 | 0.68 |
| FH3078 | 23 | 44 | 4 | 0.18 | 0.32 |
| REN02P03 | 23 | 50 | 3 | 0.11 | 0.25 |
| REN113M13F | 23 | 50 | 4 | 0.25 | 0.38 |
| FH2261 | 24 | 48 | 6 | 0.45 | 0.67 |
| FH3287 | 24 | 46 | 12 | 0.45 | 0.85 |
| REN228J19 | 24 | 49 | 3 | 0.18 | 0.36 |
| REN272I16 | 24 | 50 | 2 | 0.01 | 0.02 |
| AHT125 | 24 | 50 | 5 | 0.36 | 0.55 |
| FH2159 | 24 | 50 | 7 | 0.44 | 0.68 |
| FH3023 | 24 | 50 | 10 | 0.38 | 0.60 |
| FH3750 | 24 | 50 | 5 | 0.35 | 0.58 |
| REN106I06 | 24 | 50 | 7 | 0.40 | 0.63 |
| FH2141 | 25 | 42 | 14 | 0.46 | 0.88 |
| FH2324 | 25 | 50 | 11 | 0.47 | 0.84 |
| FH3245 | 25 | 50 | 10 | 0.44 | 0.82 |
| FH3327 | 25 | 43 | 13 | 0.49 | 0.84 |
| FH3627 | 25 | 50 | 8 | 0.41 | 0.76 |
| FH4027 | 25 | 46 | 16 | 0.45 | 0.89 |
| REN54E19 | 25 | 50 | 4 | 0.23 | 0.32 |
| DGN10 | 26 | 50 | 7 | 0.38 | 0.77 |
| FH2130 | 26 | 49 | 10 | 0.47 | 0.77 |
| FH3426 | 26 | 49 | 8 | 0.48 | 0.77 |
| *C26.733 | 26 | 49 | 4 | 0.10 | 0.18 |
| DTR26.9 | 26 | 47 | 3 | 0.23 | 0.43 |
| REN62M06 | 26 | 48 | 5 | 0.25 | 0.46 |
| FH2289 | 27 | 47 | 9 | 0.35 | 0.61 |
| PEZ16 | 27 | 48 | 7 | 0.41 | 0.68 |
| LE1002 | 27 | 44 | 7 | 0.50 | 0.78 |
| FH3221 | 27 | 49 | 9 | 0.42 | 0.76 |
| FH3924 | 27 | 47 | 5 | 0.32 | 0.59 |
| PEZ6 | 27 | 48 | 6 | 0.41 | 0.63 |
| REN181L14 | 27 | 48 | 5 | 0.44 | 0.74 |
| REN72K15 | 27 | 48 | 9 | 0.48 | 0.80 |
| C28.176 | 28 | 50 | 7 | 0.40 | 0.70 |
| FH2208 | 28 | 39 | 8 | 0.42 | 0.78 |
| FH2585 | 28 | 49 | 9 | 0.44 | 0.79 |
| REN146G17 | 28 | 50 | 4 | 0.32 | 0.47 |
| REN51I12 | 28 | 43 | 5 | 0.35 | 0.70 |
| FH1007 | 29 | 48 | 4 | 0.23 | 0.39 |
| FH2364 | 29 | 47 | 8 | 0.49 | 0.78 |
| FH2952 | 29 | 45 | 12 | 0.42 | 0.82 |
| REN45F03 | 29 | 48 | 4 | 0.38 | 0.54 |
| REN52D08 | 29 | 48 | 3 | 0.28 | 0.37 |
| FH3053 | 30 | 48 | 5 | 0.26 | 0.50 |
| FH3489 | 30 | 47 | 10 | 0.44 | 0.83 |
| FH3632 | 30 | 42 | 9 | 0.38 | 0.74 |
| FH2290 | 30 | 49 | 8 | 0.36 | 0.67 |
| REN248F14 | 30 | 50 | 3 | 0.20 | 0.34 |
| REN51C16 | 30 | 50 | 4 | 0.38 | 0.56 |
| REN89K14 | 30 | 50 | 3 | 0.26 | 0.46 |
| FH2712 | 31 | 50 | 4 | 0.32 | 0.57 |
| REN110K04 | 31 | 50 | 6 | 0.42 | 0.72 |
| REN43H24 | 31 | 50 | 5 | 0.34 | 0.68 |
| FH2189 | 31 | 49 | 8 | 0.33 | 0.64 |
| FH2239 | 31 | 46 | 13 | 0.47 | 0.87 |
| REN109B10 | 31 | 50 | 2 | 0.14 | 0.28 |
| RVC11 | 31 | 50 | 2 | 0.26 | 0.36 |
| AHT127 | 32 | 45 | 4 | 0.33 | 0.54 |
| FH2875 | 32 | 46 | 4 | 0.28 | 0.64 |
| FH3294 | 32 | 40 | 13 | 0.49 | 0.87 |
| REN244E04 | 32 | 50 | 4 | 0.39 | 0.55 |
| CPH2 | 32 | 48 | 5 | 0.45 | 0.65 |
| FH3236 | 32 | 48 | 6 | 0.26 | 0.43 |
| FH3635 | 32 | 49 | 10 | 0.35 | 0.72 |
| FH2165 | 33 | 44 | 16 | 0.49 | 0.89 |
| FH2361 | 33 | 47 | 6 | 0.38 | 0.67 |
| FH2790 | 33 | 48 | 4 | 0.38 | 0.57 |
| FH3608 | 33 | 44 | 7 | 0.34 | 0.64 |
| REN186B12 | 33 | 49 | 3 | 0.22 | 0.36 |
| FH2377 | 34 | 49 | 3 | 0.24 | 0.46 |
| FH3721 | 34 | 50 | 7 | 0.27 | 0.50 |
| FH3836 | 34 | 47 | 8 | 0.40 | 0.79 |
| REN109L16 | 34 | 49 | 2 | 0.27 | 0.37 |
| REN174M24 | 34 | 49 | 4 | 0.04 | 0.15 |
| REN243O23 | 34 | 49 | 7 | 0.39 | 0.69 |
| REN314H10 | 34 | 49 | 2 | 0.15 | 0.25 |
| FH3570 | 35 | 30 | 10 | 0.35 | 0.77 |
| REN112C08 | 35 | 50 | 3 | 0.20 | 0.44 |
| REN282I22 | 35 | 49 | 4 | 0.29 | 0.48 |
| REN94K23 | 35 | 50 | 6 | 0.37 | 0.68 |
| DTR36.3 | 36 | 46 | 8 | 0.32 | 0.68 |
| FH2611 | 36 | 50 | 9 | 0.45 | 0.83 |
| FH3865 | 36 | 46 | 13 | 0.45 | 0.85 |
| REN106I07 | 36 | 50 | 5 | 0.40 | 0.68 |
| REN179H15 | 36 | 49 | 6 | 0.38 | 0.69 |
| FH2532 | 37 | 46 | 10 | 0.45 | 0.79 |
| FH3272 | 37 | 47 | 15 | 0.45 | 0.83 |
| FH3449 | 37 | 49 | 8 | 0.44 | 0.76 |
| H10101 | 37 | 46 | 3 | 0.33 | 0.50 |
| REN67C18 | 37 | 49 | 6 | 0.36 | 0.72 |
| FH2766 | 38 | 50 | 3 | 0.14 | 0.22 |
| FH3399 | 38 | 47 | 11 | 0.48 | 0.83 |
| REN02C20 | 38 | 50 | 4 | 0.25 | 0.44 |
| REN164E17 | 38 | 50 | 6 | 0.33 | 0.70 |
