# Supplementary material for: Congenital Sensorineural Deafness in Australian Stumpy-Tail Cattle Dogs Is an Autosomal Recessive Trait That Maps to CFA10
Source: PLoS One. 2010 Oct 12;5(10):e13364. doi: 10.1371/journal.pone.0013364 (PMC2953516; doi:10.1371/journal.pone.0013364)
Supplement: Table S2 — List of primers used in fine mapping of the deafness locus on CFA10. Further details of these primers are available at (http://www.vgl.ucdavis.edu/dogset/). (0.05 MB DOC) [file pone.0013364.s003.doc]

**Supplementary Table 2**: List of primers used in fine mapping of the deafness locus on CFA10. Further details of these primers are available at (<http://www.vgl.ucdavis.edu/dogset/>).

| 10_036F_CTF |
| --- |
| 10_040A_GATAF |
| 10_042B_CAF |
| 10_043A_CTF |
| 10_044A_CAAAF |
| 10_046C_CAF |
| 10_049D_TAAAF |
| 10_051G_CAF |
| 10_025F_CTF |
| 10_028B_TAAAF |
| 10_039B_CAF |
| 10_041B_GAAAF |
| 10_048B_CAF |
| 10_048J_CAF |
| 10_050B_CTF |
| 10_052G_CAF |
| 10_027A_CTF |
| 10_029J_CAF |
| 10_032B_TAAAF |
| 10_034D_CTF |
| 10_045B_CAF |
| 10_050M_CAF |
| 10_053H_CAF |
| 10_029A_GATAF |
| 10_035G_CAF |
| 10_054G_CTF |
| 10_031A_CTF |
| 10_033F_CAF |
| 10_037E_CTF |
| 10_044G_CAF |
| 10_025F_CTR |
| 10_027A_CTR |
| 10_028B_TAAAR |
| 10_029A_GATAR |
| 10_029J_CAR |
| 10_031A_CTR |
| 10_032B_TAAAR |
| 10_033F_CAR |
| 10_034D_CTR |
| 10_035G_CAR |
| 10_036F_CTR |
| 10_037E_CTR |
| 10_039B_CAR |
| 10_040A_GATAR |
| 10_041B_GAAAR |
| 10_042B_CAR |
| 10_043A_CTR |
| 10_044A_CAAAR |
| 10_044G_CAR |
| 10_045B_CAR |
| 10_046C_CAR |
| 10_048B_CAR |
| 10_048J_CAR |
| 10_049D_TAAAR |
| 10_050B_CTR |
| 10_050M_CAR |
| 10_051G_CAR |
| 10_052G_CAR |
| 10_053H_CAR |
| 10_054G_CTR |
