# Supplementary material for: Congenital Sensorineural Deafness in Australian Stumpy-Tail Cattle Dogs Is an Autosomal Recessive Trait That Maps to CFA10
Source: PLoS One. 2010 Oct 12;5(10):e13364. doi: 10.1371/journal.pone.0013364 (PMC2953516; doi:10.1371/journal.pone.0013364)
Supplement: Table S3 — PCR primers used for amplification of the Sox10 gene. Primers were from the DOGSET of UC Davis (http://www.vgl.ucdavis.edu/dogset/) with the exception of SOX10_EXO4bmF which was designed using Primer3.0 (http://frodo.wi.mit.edu/primer3/). (0.06 MB DOC) [file pone.0013364.s004.doc]

**Supplementary Table 3:** PCR primers used for amplification of the *Sox10* gene. Primers were from the DOGSET of UC Davis (<http://www.vgl.ucdavis.edu/dogset/>) with the exception of SOX10_EXO4bmF which was designed using Primer3.0 <http://frodo.wi.mit.edu/primer3/>.

| Location of Fragment in Predicted Gene | Size bp ** | Forward Primer Name | Forward Primer Sequence | Reverse Primer Name | Reverse Primer Sequence | PCR details |
| --- | --- | --- | --- | --- | --- | --- |
| Promoter a | 600 | SOX10_PR_AF | tgccgttcacctgtatttcg | SOX10_PR_AR | ccatttcctctcccctttcc | QIAGEN Hotstar  Hifidelity buffer and Taq, 50 C |
| Intron surrounding Exon 1 | 456 | SOX10_PR_BF | cctccatacccaccctctga | SOX10_PR_BR | aggcagaggcagctggatag | QIAGEN HotStar Taq  55 C |
| Intron following exon 1 | 516 | SOX10_PR_CF | ccctcatttgtgctcgtgtc | SOX10_PR_CR | tccttcatccccaacactca | QIAGEN Hotstar  Hifidelity buffer and Taq 55 C |
| Intron surrounding Exon 2 | 427 | SOX10_PR_DF | gcccaatttccctgtaagca | SOX10_PR_DR | ggaagtggaaaaccgtgtcc | QIAGEN Hotstar  Hifidelity buffer and Taq 55 C |
| Exon 3 fragment 1 | 594 | SOX10_EX03AF | accaggtggtgggaagacag | SOX10_EX03BR | actcccccaggaagaagctc | Kapabiosystems  KAPA2G Robust Hotstart taq  56 C |
| Exon 3  Fragment 2 | 900 | SOX10_EXO4bmf | catggctcctggggttaag | SOX10_EX04R | ccatcagccacttccatcag | Kapabiosystems  KAPA2G Robust Hotstart Taq  58 C |
| Intron surrounding exon 4 | 900 | SOX10_EXO4bmF | catggctcctggggttaag | SOX10_EX04R | ccatcagccacttccatcag | Hotstar  Hifidelity buffer and Taq 55 C |
| Intron surrounding exon 5 | 1140 | SOX10_EX05AF | ccagccctagcccattacag | SOX10_EX05CR | cttctcctctgcccagccta | HotStar Taq  55 C |
